# Acupuncture for mild cognitive impairment in elderly people: Systematic review and meta-analyses

**DOI:** 10.1097/MD.0000000000022365

**Published:** 2020-09-25

**Authors:** Weitong Li, Qing Wang, Shizheng Du, Yalou Pu, Guihua Xu

**Affiliations:** a School of Nursing, Nanjing University of Chinese Medicine; b Nursing Department, Jiangsu Cancer Hospital, Nanjing, Jiangsu, PR China.

**Keywords:** acupuncture, meta-analysis, mild cognitive impairment

## Abstract

**Background:**

Acupuncture has an unique role in preventing and managing mild cognitive impairment (MCI) in nonpharmaceutical therapies because of its small wound, mild pain, and high security for many years. However, there is no systematic review evaluating safety and efficacy of acupuncture for MCI in elderly people. Therefore, this study will provide a protocol to explore the effectiveness and safety of acupuncture for MCI in the elderly.

**Methods:**

Retrieval from 8 electronic databases was conducted to determine eligible trials published until May, 2019. Homogeneity qualified studies were included for data were extracted such as study country location, demographic characteristics, and measure outcomes, and were analyzed by a random effect model and sensitivity analyses to identify heterogeneity. Review Manager (Revman Version 5.3) software will be used for data synthesis, sensitivity analysis, meta regression, subgroup analysis, and risk of bias assessment. A funnel plot will be developed to evaluate reporting bias.

**Results:**

A total of 15 randomized control trials involving 1051 subjects were included. The results were as follows: Compared with the control group, the clinical efficacy rates of acupuncture was better, odds ratio = 2.52, 95% confidence interval (CI) (1.86, 3.42), *P* < .00001, mini-mental state examination scores (mean difference [MD] = 1.53, 95% CI [1.04, 2.01], *P* < .00001), Montreal cognitive assessment scores (MD = 2.05, 95% CI [1.17, 1.92], *P* < .00001), activity of daily living scale (MD = 1.71, 95% CI [–1.38, 4.79], *P* > .05), and clock drawing task scores (MD = 1.91, 95% CI [1.74, 2.08], *P* < .00001).

**Conclusion:**

This study shows that acupuncture is beneficial for improving aspects of cognitive function in elderly people with MCI, which suggests that acupuncture may be an effective alternative and complementary approach to existing therapies for elderly people. More rigorous experimental studies and longer follow-up studies should be conducted in the future.

## 1 Introduction

Mild cognitive impairment (MCI) is an intermediary state between normal aging and clinical Alzheimer disease (AD). The elderly people with MCI are at increased risk for conversion to clinical AD.^[1–3]^ People with MCI have significant impairment in all 5 cognitive domains, namely, speed/attention, memory and learning, visual spatial function, language and executive function.^[4]^ Approximately 5% to 10% of the cases with MCI will evolve into dementia per year.^[5–7]^ A Canadian study demonstrated that almost half of people with MCI will progress to fulfill the diagnostic criteria of dementia in 5 years.^[8]^ Early intervention and treatment in the MCI state can reduce the incidence of AD and delay or prevent the development of the disease.^[9,10]^ Once MCI develops into AD, severe cognitive decline will have a huge impact on people’ daily lives and their family.^[11,12]^ No approaches have been developed specifically to prevent and treat MCI. There are ongoing randomized control trial (RCT) studies but acetilcholinesterasis inhibitors are not approved for MCI. Long-term use of drugs may cause nausea, vomiting, bradycardia, increased gastric acid secretion, and even worsen cognitive impairment and adverse reactions. More and more other effective measures have been given attention.

In recent years, the “Chinese guidelines for the diagnosis and treatment of dementia and Cognitive Disorder 2015”^[13]^ suggested a strategy for the prevention and treatment of MCI involving reducing the conversion rate from MCI to AD. A number of basic and clinical studies have provided evidence that acupuncture is beneficial for the treatment of dementia or MCI.^[14–19,21]^ The forms of acupuncture include simple acupuncture,^[18–22]^ acupuncture combined with western medicine,^[25,26]^ acupuncture combined with cognitive function training,^[23–29]^ acupuncture combined with moxibustion,^[32]^ acupoint embedding, auricular compression combined with herbs^[33]^ and acupoint stimulation.^[34]^ Acupuncture has an unique role in preventing and managing MCI in nonpharmaceutical therapies because of its small wound, mild pain, and high security for many years. Several systematic reviews and meta-analyses have been published to discuss the role of acupuncture in treating MCI.^[35–37]^ However, in the existing meta-analyses, there was no systematic review or meta-analyses has been performed to evaluate the effectiveness of acupuncture specifically for elderly people, most samples in the RCTs included people under 60 years of age. These previous studies were typically meta-analyzed at final time points, paying no attention to all time points of repeated measures. The original study of systematic evaluation had a relatively large age range and was not analyzed and compared specifically for the elderly. Compared with previous systematic reviews, this study has added the literature that has been updated in recent years. Therefore, this study planned to conduct a systematic review and meta-analyses to evaluate the evidence from all available RCTs that evaluate acupuncture on MCI in elderly people, which could provide constructive guidance for clinical medical staff.

The structure of the whole article consisted of 5 parts, including Introduction, Methods, Results, Discussion, and Conclusion. In the results section, this study analyzed the efficacy of acupuncture in the 5 major aspects of Clinical efficacy rates, mini-mental state examination (MMSE) scale score, Montreal cognitive assessment (MoCA) test score, activity of daily living scale (ADL) scale score and clock drawing task (CDT), and conducted subgroup analysis based on different course of treatment. This study summarized results and innovations in the discussion and conclusion section.

## 2 Methods

This systematic review and meta-analysis has been reported in accordance with the Preferred Reporting Items for Systematic Reviews and Meta-Analyses recommendations. The trial registration number is as follows: PROSPERO registration no. CRD42019120033. All analyses were based on previous published studies, thus no ethical approval and patient consent are required.

### 2.1 Selection strategy

This study searched the following electronic databases to identify eligible trials published from inception to May 1, 2019: including PubMed, Web of science, Cochrane Library, EMBASE, CBM, Chinese National Knowledge Infrastructure Database, VIP Database, and Wanfang Database. This study used the following medical subject heading terms and text words when searching: (acupuncture OR meridian OR acupuncture treatment OR acupuncture therapy OR acupuncture points OR electroacupuncture OR ear acupuncture OR fire needle OR bee needle) AND (mild cognitive impairment OR MCI OR cognitive dissonance OR delirium OR dementia OR amnestic OR cognitive disorders) AND (elderly OR old people OR older people OR aged OR old population) AND (randomized controlled trial OR “random^∗^” OR “alloc^∗^” OR “assign^∗^”). Accordingly, this was the search strategy used in the 8 databases.

### 2.2 Study selection

The inclusion criteria were as follows:

This study included MCI elderly people who were clearly diagnosed by domestically and internationally recognized criteria, diagnostic criteria included:

(1) Diagnostic criteria for MCI in the American Psychiatric Manual of Psychiatry and Statistics, 4th Edition (DSM-IV)^[38]^;
(2) MCI Clinical Diagnostic Standards revised by Petersen et al 2001^[39]^;
(3) MCI Clinical Diagnostic Standards revised by Petersen et al 1999^[40]^;
(4) The Reference Standard of Deficiency Syndrome Differentiation in traditional Chinese Medicine^[41]^;
(5) The 2006 Chinese expert consensus on cognitive dysfunction^[42]^;

Furthermore, these studies should meet the following inclusion criteria (PICO format).

**P** (population): Studies that examined elderly people suffering from MCI which caused by nonorganic diseases. This study included the studies that focused on elderly participants. According to the World Health Organization, the elderly population is referred to as 60+ years old (World Health Organization).

**I** (intervention): People in the treatment group must have received acupuncture therapy, either is used alone or in combination with other therapies. In acupuncture therapy, acupoints can be head points, ear points, experience points or points outside the meridian; Acupuncture methods can be traditional acupuncture needle or electroacupuncture. However, acupuncture therapy does not include other acupuncture methods, such as finger massage acupoints, laser acupuncture, magnetic pole acupuncture, and acupoint injection. The specifications of needle and electroacupuncture instruments are not limited to manufacturers because there is no international standard at present. Moreover, acupuncture can be combined with other therapies (such as basic treatment, medication, and cognitive training therapy), and acupuncture techniques and points do not distinguish. Acupuncture can be combined with the same basic therapy as that of the control group simultaneously.

**C** (comparison): The control group needed to have received drug therapy (Donepezil/Nimodipine/Yinao capsule/Duxil/Oxiracetam Injection) and psychological intervention (cognitive training).

**O** (outcome): The outcome measurements needed to include at least 1 authority scale of cognitive assessment such as the MoCA, MMSE, CDT, ADL, considering the heterogeneity of outcomes, 15 RCTs used different outcome indicators to analyze the validity of acupuncture, this study excluded the literature of self-designed outcome indicators. Finally, this study chose 5 indicators as the main outcome indicators which included the clinical efficacy rates, the MoCA, MMSE, CDT, ADL. This study also included grey literature in the evaluation in the form of 3 degree papers.^[28–30]^ There are no restrictions on the language or type of publication.

The exclusion criteria are as follows:

(1) no control group or multiple control groups;
(2) nonclinical trials (reviews, case and repeated reports, expert experience reports, meeting notices, and nonrelated contributions to research);
(3) animal experiments;
(4) literature on treatment mechanisms;
(5) literature on acupuncture was also included in the basic treatment of the control group;
(6) self-control trials;
(7) self-designed curative effect index;
(8) documents in which articles or data were published repeatedly.

### 2.3 Data extraction

The details of included trials were extracted independently by 2 authors (LWT and WQ) using a standard data extraction form, which included the following items:

(1) the basic characteristics of the included trials: title, authors, publication year, literature sources, country where the trial was conducted, publication language, sample size, diagnosis standard, study design, interventions, controls, treatment duration, and adverse events; and
(2) the basic characteristics of the included subjects: age, gender, intervention, course of treatment, diagnostic criteria, inclusion criteria, exclusion criteria, outcome measures.

During this process, any disagreements between the 2 reviewers were resolved through consensus, and the discrepancy was resolved by discussion or judged by the third author (DSZ).

### 2.4 Quality assessment

The quality of the selected studies was assessed using the RCT-quality criteria recommended in the Cochran intervention system Review Manual, 5.1.0 (Higgins and Green 2011).^[43]^ These criteria include 6 items on sequence generation, allocation concealment, blind, incomplete data results, selective result reporting, and other biases (baseline imbalances, early discontinuities, and sources of funding). Each project was rated as a “low deviation risk,” “unclear deviation risk” or “high deviation risk” and treated as level “A, B, and C.”^[43]^ Because acupuncture treatment is unlikely to be double blind, this study concluded that single-blind outcome evaluators were “consistent” with blinding. In general, this study considered randomization, distributive concealment, blind, incomplete data results, selective outcome reporting, and trials of other potential sources of bias. If the content of the study is insufficient to determine the risk of bias, the author of the study should be contacted for further information.

### 2.5 Data synthesis and statistical analysis

RevManV.5.3 (Cochrane Collaboration) was the tool of statistical analysis. The effect estimates for the dichotomous data were presented as odds ratios (ORs) with their 95% confidence intervals (CIs), and the continuous data were presented as the mean differences (MDs) and their 95% CIs. In each meta-analyses, the standard *χ*^2^ statistics and *I*^2^ test were used to measure the statistical heterogeneity (Higgins and Green, 2011).^[44]^ A fixed-effect model was adopted if no significant heterogeneity existed (*I*^2^ < 50%); a random-effect model was adopted if significant heterogeneity existed. Publication bias was assessed through funnel plots. Subgroup analysis were performed if the primary outcome demonstrated statistically significant differences between the 2 groups.

## 3 Results

### 3.1 Search process

**Figure 1:**
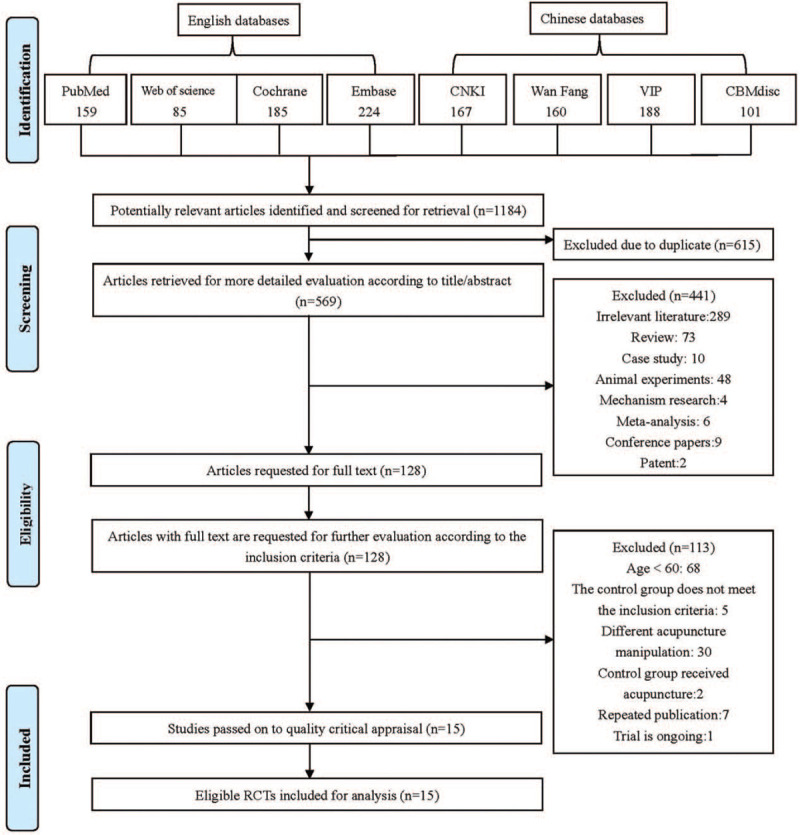
Flow chart of trial selection process.

The results of the search process are shown in Figure 1. A total of 1184 articles of potential relevance were retrieved from 8 databases, of which 615 were duplicates. After a preliminary screening of the titles and abstracts, 441 studies were excluded for the following reasons: 289 were unrelated articles, 73 were reviews, 10 were case studies, 48 were animal experiments, 4 were mechanism studies, 6 were meta-analyses, 9 were conference papers and 2 were patents. Overall, 128 RCTs were obtained for a full-text assessment. These retrieved articles were subsequently evaluated by deep reading. Finally, 15 studies^[18–32]^ were ultimately included in the qualitative synthesis and meta-analyses.

### 3.2 Methodological quality assessment

**Figure 2:**
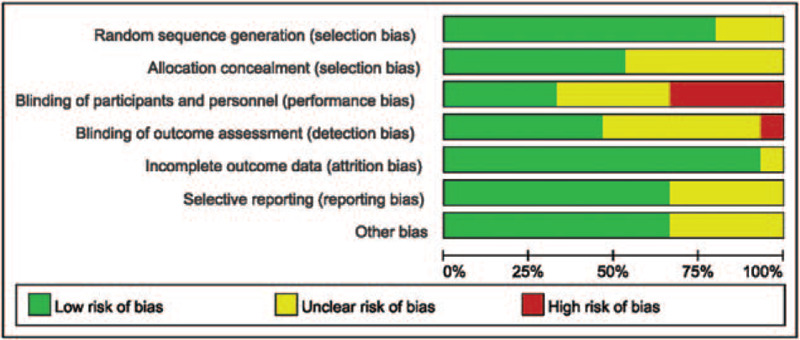
Risk of bias graph of included trials.

**Figure 3:**
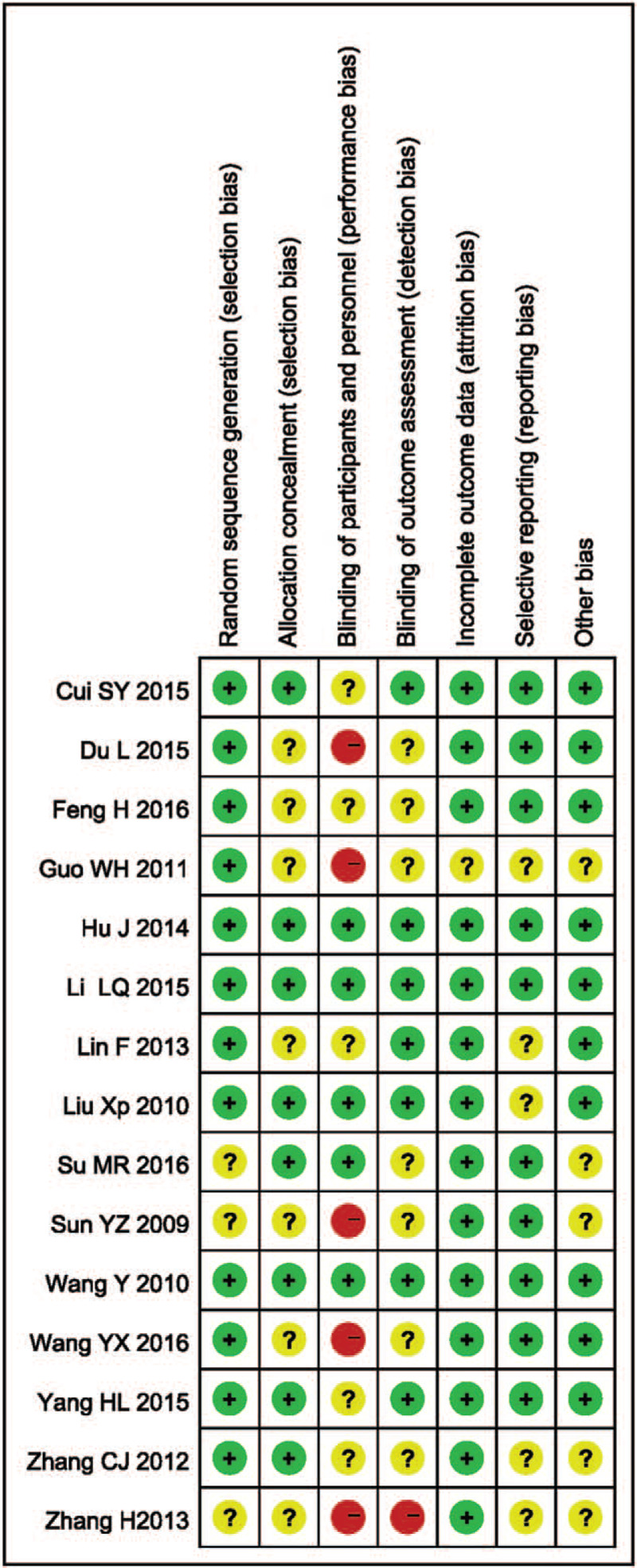
Risk of bias summary of included trials.

The risk of bias assessment of each included trial is summarized in Table 1, the risk of bias graph is shown in Figure 2, and the risk of bias summary is shown in Figure 3. Based on the Cochrane manual, the quality is divided. RCT was divided into 3 levels (A, B, and C). “A” means that all or most of the 6 criteria were met, which represents a low risk bias. For candidate RCTs, if 1 or more criteria partially meet the B rating, it represents an unclear risk bias. However, if 1 or more criteria are insufficient, “C” is the high risk of bias level. The outcomes of methodological quality of all included studies are listed in Table 1.

**Table 1:**
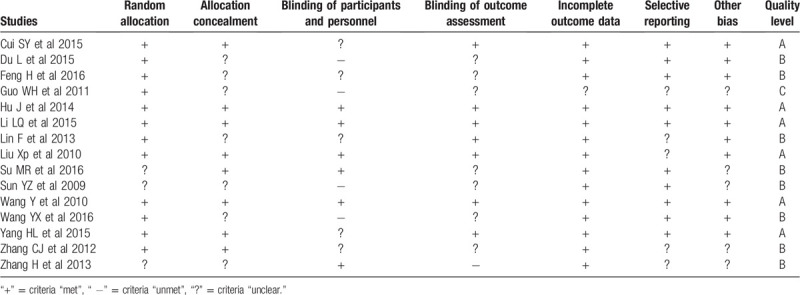
Quality scores of trials included in this review.

Fifteen RCTs^[18–32]^ were considered to be eligible for analysis. The information concerning their characteristics is summarized in Table 2. Because the concept of MCI was put forward later, and its connotation has been revised,^[45]^ there are relatively few studies on MCI, and its diagnosis has not yet arrived at a standard. Thus, this study excluded the studies that have developed their own criteria for inclusion and exclusion and studies in which the diagnostic criteria met the 6 criteria mentioned in the first half of this article. Generally, the 15 included RCTs were published between 2009 and 2016. The total number of MCI elderly participants was 1051, with 530 people allocated to the acupuncture group (experimental group) and 521 people allocated to receive a conventional treatment (control group), which included western medicine alone (eg, 1 trial^[30]^ directly compared acupuncture with Nimodipine, 3 trials^[18–20]^ compared acupuncture with Donepezil). Eight trials examined the role of acupuncture as an adjunct to Nimodipine,^[25,26,30,32]^ Duxil,^[24]^ Oxiracetam Injection,^[27]^ and the traditional Chinese medicine Yinao capsule alone,^[22]^ where the people in both groups received medical therapy and were randomized to receive additional acupuncture or not. Two trials^[23,29]^ set cognitive training as a control group, while the experimental group was a combination of acupuncture and cognitive training. The number of participants in each experimental group of study varied from 9 to 80. In these 15 studies, the course of treatment time ranged from 30 days to 3 months, this study categorized these durations as short-term (up to 1 month), medium-term (2 months) or long-term (3 months); each treatment session lasted 30 minutes. Overall, 15 RCTs treated GV 20, EX-HN 1, ST 2, GB 20, GB12, BL 10, HT 7, PC 6, GV 26, SP 6, LR 3, ST 40 as the main acupoints. The outcome measures included the clinical efficacy rate, MoCA, MMSE, CDT, ADL, gobal deterioration scale, clinical dementia rating scale, clinical memory scale, event-related potential-P300, revised Hasegawa dementia scale, modified Barthel and scale for the differentiation of syndromes in vascular dementia and memory quotient. The clinical efficacy rate of all studies was consistent.

**Table 2:**
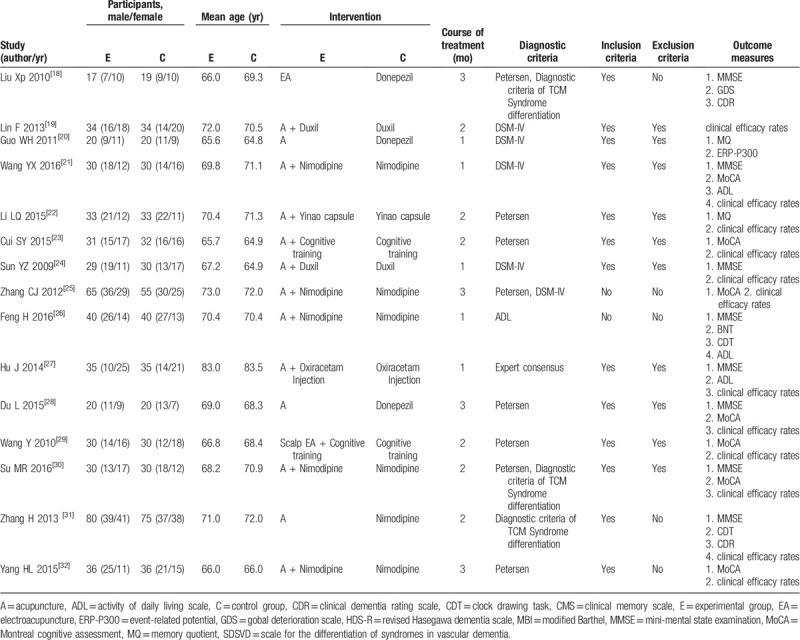
Characteristics of the included studies.

### 3.3 Clinical efficacy rates

**Figure 4:**
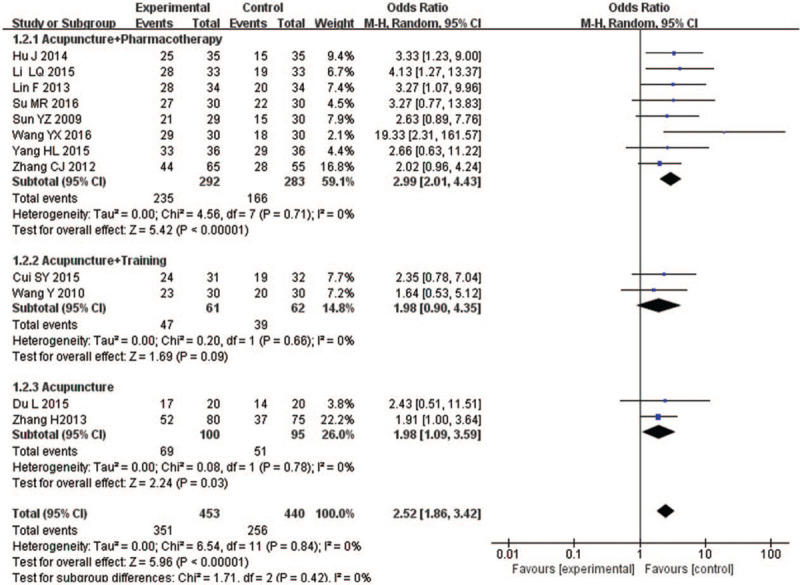
Forest plot of comparisons by different forms of acupuncture versus routine treatment for the outcome of the clinical efficacy rates.

This analysis included 12 articles, which included the outcome indicators of the clinical efficacy rates. According to different forms of acupuncture (acupuncture + pharmacotherapy, acupuncture + training therapy, acupuncture), this study made a subgroup analysis. According to Figure 4, the clinical efficacy rates was significantly improved in the treatment group (OR = 2.52, 95% CI [1.86, 3.42], *Z* = 5.96, *P* < .0001), competed with the control group of 12 studies (n = 256) with low heterogeneity (*I*^2^ = 0%). The result showed that acupuncture combined with pharmacotherapy or acupuncture treatment alone have better efficacy on elderly people with MCI, but the effect of acupuncture combined with training therapy is not significant (OR = 1.98, 95% CI [0.90, 4.35]), *Z* = 1.69, *P* = .09). This not only showed that acupuncture has a therapeutic effect on MCI in elderly people, but also acupuncture can better promote the absorption of drugs, making the curative effect more significant.

### 3.4 MMSE scale score

**Figure 5:**
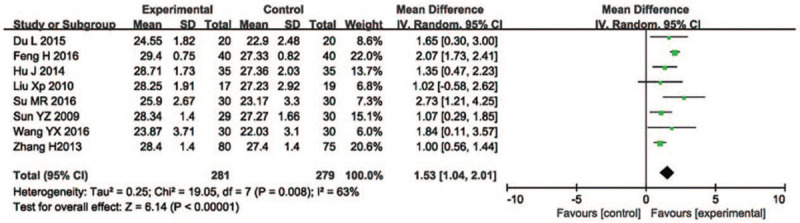
Forest plot of comparison of acupuncture versus routine treatment for the outcome of the MMSE scale score. MMSE = mini-mental state examination.

Eight studies involving 281 participants in the treatment group and 279 in the control group assessed the MMSE scale score. According to Figure 5, the data heterogeneity test *I*^2^ = 63%, when *I*^2^ > 25% and 50% showed moderate heterogeneity, and *I*^2^ ≤ 70% could still be used for the meta-analyses; thus, this study did the sensitivity analysis, the forest plot showed that the *I*^2^ dropped from 63% to 0%, and the article that this study removed in the sensitivity analysis had repeated interventions compared with the other RCTs, this study could not eliminate this article in the end, so the random effects model was used. The results of the meta-analyses showed that acupuncture therapy could improved the MMSE scores compared with the control group (MD = 1.53, 95% CI [1.04, 2.01], *Z* = 6.14, *P* < .0001).

### 3.5 MoCA test score

**Figure 6:**
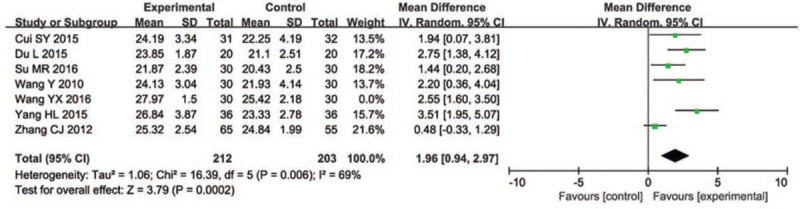
Forest plot of comparison of acupuncture versus routine treatment for the outcome of the MoCA test score. MoCA = Montreal cognitive assessment.

The MoCA test score was almost the same in the treatment group (MD = 1.96, 95% CI [0.94, 2.97], *P* = .0002), compared with people who did not have the acupuncture therapy of 7 studies (n = 415) with high heterogeneity (*I*^2^ = 69%). When this study used a fixed-effect model, heterogeneity (*I*^2^ = 69%). Given *I*^2^ > 50%, this study employed the randomized-effect model and the *I*^2^ still was 69%, the result showed that compared with the control group, the MoCA test score was significantly higher in the acupuncture group (Fig. 6). Forms of acupuncture of eligible RCTs included acupuncture combined with western medicine or cognitive training, only 1 study used acupuncture alone as a treatment. The durations of treatment and intervention methods were different, which is the reason for the heterogeneity.

### 3.6 ADL scale score

**Figure 7:**
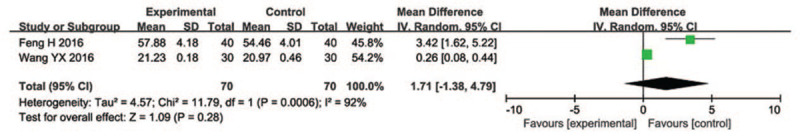
Forest plot of comparison of acupuncture versus routine treatment for the outcome of the ADL scale score. ADL = activity of daily living scale.

Two studies involving 70 participants in the treatment group and 70 in the control group assessed the ADL scale score (MD = 1.71, 95% CI [–1.38, 4.79], *P* > .05). The data heterogeneity test *I*^2^ = 92%, *I*^2^ > 70% could not be used for the meta-analyses, so the result showed that the treatment group was combined with acupuncture therapy would not have the improvement in ADL scale score (Fig. 7).

### 3.7 CDT

**Figure 8:**
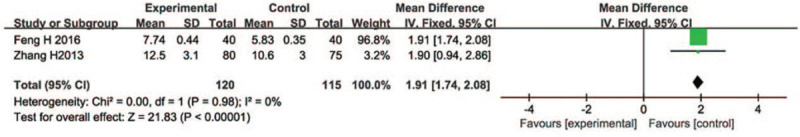
Forest plot of comparison of acupuncture versus routine treatment for the outcome of the CDT score. CDT = clock drawing task.

Two studies using CDT score showed that the treatment group had the obvious effects in MCI elderly people with no heterogeneity (*I*^2^ = 0%) (MD = 1.91, 95% CI [1.74, 2.08], *Z* = 21.83, *P* < .0001), which illustrated that in the future treatment of MCI in the elderly, CDT could be used to detect and exercise the cognitive level of the elderly, while instructing the elderly to carry out the task of clock painting, medical staff could purposefully exercise the memory of the elderly (Fig. 8).

### 3.8 Course of treatment

**Figure 9:**
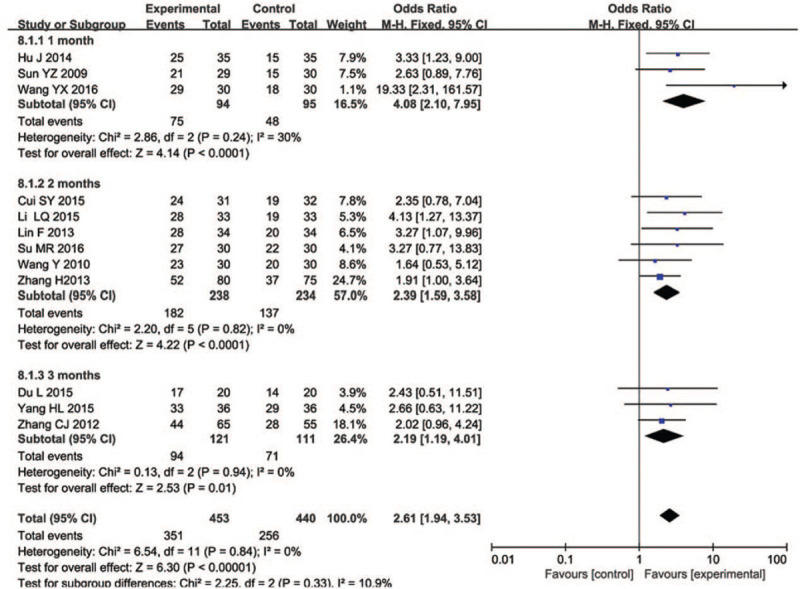
Forest plot of comparisons by different duration for the outcome of the clinical efficacy rates.

This study did the meta-analyses with different subgroups including 1 months, 2 months, and 3 months (Fig. 9). This analysis included 12 studies with 453 participants (treatment group) and 440 elderly people (control group). The heterogeneity of the 1 month was greater (*I*^2^ = 30%) and the reliability of the results was lower. The review demonstrated that 2 months (*I*^2^ = 0%, MD = 2.39, 95% CI [1.59, 3.58]) and 3 months (*I*^2^ = 0%, MD = 2.19, 95% CI [1.19, 4.01]) of acupuncture treatment could have better efficacy in clinical efficacy rates, so longer treatment should be implemented in the future to facilitate a better comparison.

### 3.9 Bias analysis

**Figure 10:**
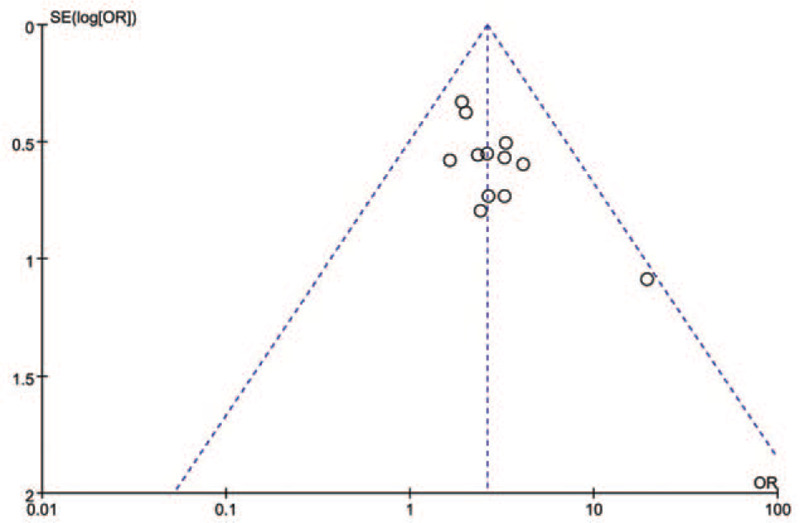
Funnel diagram of the treatment in elderly people with MCI. MCI = mild cognitive impairment.

This study performed the funnel diagram to test whether there was a published bias in this study (Fig. 10). With the combined OR value (the dashed line in the diagram) as the center, the scattered points of the 12 RCTs were well distributed, and the results of the small sample study were roughly distributed around the global effect (dashed line) or large sample study. Based on the shape of an funnel diagram and basic symmetry, the results showed that the bias of the 12 study samples was not significant.

### 3.10 Adverse events

Eight trials showed that they did not have the exfoliated specimen, and only 1 trial did not mention the standard of the exfoliated specimen, the other remaining 7 articles, 3 trials mentioned that the reason for exfoliating was that the participants could not endure acupuncture pain. One RCT mentioned the specific safety tests that included the general examination items of body temperature, blood pressure, pulse, breathing, blood routine, urine routine, and stool routine. One RCT mentioned the examination before and after the administration of medicine and the following steps:

(1) a neurological examination and general physical examination;
(2) a laboratory examination to check routine blood, urine, stool, liver and kidney function, blood glucose, blood lipids, and so on;
(3) an electrocardiogram.

## 4 Discussion

### 4.1 Summary of evidence

This systematic review and meta-analyses illustrated that acupuncture and its combined therapy were more effective than conventional therapy alone for MCI in older people in effectiveness, as indicated specifically by increases in the living ability state and mental cognitive state of the elderly, especially the MMSE scale score (MD = 1.53), MoCA test score (MD = 1.96), ADL scale score (MD = 1.71), and CDT (MD = 1.91). In the subgroup analysis, the review demonstrated that 2 months of acupuncture treatment could have better efficacy in clinical efficacy rates. Sensitivity analysis showed that the results of this meta-analyses were robust.

Several previous systematic reviews and meta-analyses have been published to explore the role of acupuncture interventions for MCI in middle-aged people. These articles were somewhat constant with each other with the conclusion that acupuncture is an effective and safe method for the treatment of MCI, but the clinical efficacy is not better than the routine treatment of western medicine.^[36,37,46,47,48,49]^ The results of this study show that acupuncture can effectively improve the MMSE and ADL scores of the elderly with MCI, which is similar to studies results of YT Yao and SZ Mao.^[46,47]^ However, this study analyzed data from 15 RCTs involving 1051 individuals older than 60 years of age to summarize and evaluate the available evidence on the efficacy and safety of acupuncture therapy for MCI, the largest subgroup analyzed different courses of treatment improved clinical efficacy rates in elderly people, this study further found that acupuncture can also improve the ADL and CDT scores of MCI elderly people. Moreover, the clinical efficacy rates in acupuncture group may be the most significant at 2 months of treatment, the interventional time is becoming more and more important, through this approach that giving us a more concrete picture on the role of acupuncture in improving the quality of life and mental state of the elderly than before.

This study also undertook subgroup analyses of different forms of acupuncture, including acupuncture and pharmacotherapy, acupuncture and training therapy, acupuncture. The result showed that acupuncture combined with pharmacotherapy or acupuncture treatment alone have better efficacy on elderly people with MCI, this not only showed that acupuncture has a therapeutic effect on MCI in elderly people, but also acupuncture can better promote the absorption of drugs, making the curative effect more significant. Acupuncture treatment has certain effects on various aspects of Clinical efficacy rates, MMSE scale score, MoCA test score, and CDT. Specifically, acupuncture is effective in the treatment of MCI in elderly people, and the acupuncture group had statistically significant better outcomes than the control group.

In order to avoid methodological weaknesses, the sample size should be expanded as much as possible, a small sample size can distort the results of meta-analyses, by over estimating treatment effects. 10 RCTs were considered to be at low risk of bias, 5 RCTs (33%) were at an unknown risk of bias. Additionally, with regard to the risk of bias, its major responsibility was the lack of proper blinding and allocation concealment. The estimate of the intervention effect can be exaggerated when there is inadequate allocation concealment or lack of blinding in trials where a subjective outcome is analyzed. These methodological weaknesses may lead to an overestimation of treatment effects.

The innovation of this paper is that this study conducted a systematic review and meta-analyses of the elderly people with MCI who received acupuncture treatment. With slighter heterogeneity of intervention among trials and people this study included in the RCTs were all elderly people, the conclusion and result may be more reliable to generalize and simplify. This study performed a subgroup analysis and a sensitivity analysis when this study encountered inconsistent conditions, all of which might have helped to decrease clinical heterogeneity to some extent. At the same time, this study chose 5 indicators as the main outcome indicators, the clinical efficacy rates and the MoCA, MMSE, CDT, and ADL scores; these 5 indicators are closely related to cognition. Accordingly, this study can explain the influence and significance of cognitive impairment for the elderly through an analysis of these indicators. This review shows that acupuncture treatment can significantly improve the various cognitive function indicators of elderly people. After implementing the acupuncture therapy strategy, in order to improve the treatment effect of the disease and reduce the adverse reactions of elderly MCI people, the acupuncture therapy should be popularized. The most important direction of future research is to carry out more diversified methods and measures for acupuncture in elderly people with MCI, to cultivate the ability and technology of medical staff in the acupuncture of elderly people with MCI and strengthen clinical education and improve the career quality of clinical physician of Chinese medicine.

### 4.2 Limitations

There are some limitations of this review. First, this study considered the commonness of acupuncture stimulation and assumed that all treatments were similar; thus, this study did not distinguish all acupuncture methods such as common acupuncture from electroacupuncture and body acupuncture from other acupuncture methods. Second, it was difficult to find out the influence of contingency factors. The operation technique used in acupuncture, the choice of acupoint, and the depth of acupuncture are not discussed in this paper. The quality of clinical trials should be considered. Third, despite the utilization of a range of outcome measures, the impact based on the smallest clinically important difference for each metric may not be reflected.

Acupuncture as a safety and economical method which has also been shown to be effective in the treatment of MCI, but this study have to take into account that the subjects of the study are people over the age of 60. Future studies could take into account the following points. First, In the future, this study hope systematic review can be updated based on more rigorous and powerful evidence. Second, optimal acupoint selection, session duration, and application frequency have not been established. As clinical health care workers, this study should use humanized treatment methods to care for the elderly. This study need to pay more attention to the mental health of the elderly, and it is more important to provide them with continuous and effective psychological care and counseling than treatment and at the same time strengthen health education for MCI in older people.

## 5 Conclusions

Preliminary evidence indicated that as a relatively safe and reliable intervention, acupuncture therapy has a significant positive effective on cognitive and memory function in elderly people with MCI, and improved the MMSE, MoCA, ADL, and CDT scores. However, the methodological quality of some trials included in this review were of low quality. Despite the apparently positive findings, it is premature to conclude the effectiveness of acupuncture for the treatment of MCI in elderly people due to the heterogeneity of the included trials and the generally low methodological quality of the included trials. Multi-center, double-blinded, and placebo-controlled RCTs are required to provide stronger evidence.

## Acknowledgment

The authors wish to thank Professor Shi-Zheng Du, School of Nursing, Nanjing University of Chinese Medicine, for giving advice on writing this manuscript.

## Author contributions

**Conceptualization:** Li Weitong.

**Data curation:** Li Weitong, Wang Qing, Pu Yalou.

**Methodology:** Du Shizheng.

**Project administration:** Xu Guihua.

**Software:** Li Weitong.

**Writing – original draft:** Li Weitong. Wang Qing, Xu Guihua.

**Writing – review & editing:** Li Weitong, Pu Yalou.
